# Effectiveness of Nurses’ Training in Identifying, Reporting and Handling Elderly Abuse: A Systematic Literature Review

**DOI:** 10.3390/geriatrics7050108

**Published:** 2022-10-01

**Authors:** Pratibha Ranabhat, Monica Nikitara, Evangelos Latzourakis, Costas S. Constantinou

**Affiliations:** 1 Department of Life and Health Sciences, School of Sciences and Engineering, University of Nicosia, Nicosia 1700, Cyprus; 2 Department of Basic and Clinical Sciences, University of Nicosia Medical School, Nicosia 2408, Cyprus

**Keywords:** nurse education, trainings, elderly abuse, systematic review

## Abstract

The elderly population globally is estimated to grow by one-third of the world’s population by the year 2050. At the same time, elder abuse and neglect have been acknowledged as major growing concerns. With the growing elderly population and increasing concerns about elder abuse, understanding the ways to deal with elder abuse is important. The healthcare professionals, especially nurses, are among the first groups who come in contact with the elderly population and can identify and assess cases of elder abuse. There is evidence to suggest that nurses lack knowledge in the assessment, identification, management, and reporting of an elder abuse case. This study aims to explore the available literature in the effectiveness of training programs for nurses in elder abuse management. The search strategy included the electronic databases CINHAL, Medline, and Health Source. A total of 646 research articles published between 2010 and 2021 were screened against inclusion and exclusion criteria. After reviewing and removing duplicates and irrelevant studies, 14 articles were included in this review. The findings of this literature review revealed that providing education and training for nurses in elder abuse can enhance their knowledge and increase identification and reporting of elder abuse cases. It also indicated that mixed teaching methods, such as face-to-face lectures, simulation, or case scenarios and debriefings or feedback can strengthen the learning process of nurses. In conclusion, educational programs for nurses can significantly improve the identification, reporting, and handling skills of elder abuse incidents. This finding can help in developing accurate strategies for minimizing and preventing elder abuse cases. From the results of this systematic review, we propose the ECLiPSE pathway for the effective training of nurses and handling of elder abuse cases, eventually contributing to decreasing the incidents.

## 1. Introduction

Every year the rate of the global elderly population group is increasing rapidly, which is the result of different innovative health care treatment that helps in the improvement of health conditions ([1]. The World Health Organisation estimated that by the year 2050 one third of the population will be 60 years old or older [2]. With this increasing life expectancy, the elderly populations are at high risk of various health and social problems, such as ischemic heart disease, cancer, respiratory problems, decreased cognitive function, and social isolation. Along with the escalating chronic disease, the elderly have an increased dependency on others for their daily living activities [3], which means that they might be at increased risk of being a victim of abuse.

According to World Health Organisation (2020) [2], elder abuse is defined as a “single or repeated act or lack of appropriate action, occurring within any relationship where there is an expectation of trust which causes harm or distress to an older person”. Along similar lines, the Centre for Disease and Prevention (2018) [4] defined elder abuse as the “intentional act or failure to act by a caregiver or another person in a relationship involving an expectation of trust that causes or creates a serious risk of harm to an older adult”. Although a more unified definition of elder abuse is still discussed, most definitions include similar components such as “a single or repeated act of commission or omission, which occurs within a relationship of trust and cause harm or distress to an older person” [5].

There are different forms of elder abuse such as physical, psychological, sexual, financial, and neglect [5]. According to Kosa et al. [6], the prevalence rate of psychological abuse is the most common, while the least noticed form of abuse was sexual. Yon et al.’s [7] systematic review indicated that the prevalence rate of psychological abuse was 11.6%, financial 6.8%, neglect at 4.2%, physical abuse at 2.6%, and sexual abuse 0.9%. However, some studies stated that physical abuse was identified and reported more by healthcare professionals [8,9]. This could be explained by the fact that the characteristics of physical abuse, which include bruises, fractures, and swelling, are more likely to be noticed by healthcare providers than by other professionals.

Elder abuse has become a continuous problem globally with increasing rate every year [5]. Around the world every six elderly, one is a victim of some kind of abuse [7]; however, only four percent report it [2]. In the United States each year one to two million elderly face some form of abuse [4]. In a report by World Health Organisation in the European region it was estimated that 4,000,000 elderly annually would face maltreatment [2]. Along similar lines, a community survey in Malaysia revealed that approximately 4.5% of older people have been victims of abuse [10]. Harries et al. (2014) [8] indicated that elder abuse annually in Queensland, Australia, was concerning, while Acierno et al. [11] showed that one in ten elderly in the United States was a victim of elder abuse. Likewise, McDonalds’ [12] study in Canada found that 8.2% of the population faced a type of abuse in their older age. Other studies have highlighted the increasing rate of elder abuse in recent years, which also has contributed to the mortality and morbidity of victims [3]. The prevalence rate accounts for 0.7% to 14%, which shows that many cases go unnoticed and unreported [8]. Interestingly, a global study identified that the prevalence rate increased from 2.2% to 36.2% and was higher in Asian countries as compared to Western countries [13]. This study found that among Asian countries, China and India had the highest incident rate with 36.2% and 14.0%, respectively. Similarly, the prevalence rate of elder abuse was higher in retirement homes as the elderly were more socially vulnerable [14]. A National Survey of Older Persons (NSOP) in 2018 in Korea revealed that the highest rates of abuse were psychological (42.9%), physical (37.3%), and neglect (4.7%) [15]. Harries et al. (2014) [8] highlighted that the majority of older adults were not willing to report if they had been abused. Harries et al. carried on explaining that the elderly who were cared by the abuser, feared for not having support in the future, while others were emotionally attached to their caregivers, especially when they were close relatives. In addition, some older people were concerned that their abuser would not be punished, and others thought some form of abuse (e.g., verbal and financial) was normal in some cultures.

Because they work closely with patients, healthcare professionals have the opportunity to identify, assess and manage elder abuse early [3,16]. They are also ethically obliged to report any suspected cases of elder abuse [17]. However, healthcare professionals face many different challenges in detecting and managing the case of elder abuse. According to the findings of several studies, most healthcare professionals might not be aware of the mandatory reporting law, age care law, signs, and symptoms of the abused [16]. An earlier study by Taylor et al. [18] found that most of the time, even though nurses were aware of mandatory reporting, they were hesitant to report cases of elder abuse. Inadequate and inappropriate knowledge on the topic of elder abuse and the normal physiological changes that occur to the elderly could be another barrier to tackle elder abuse. In support, Corbi et al.’s [19] study of European healthcare professionals showed that healthcare professionals’ knowledge at the European level was rather limited. Similarly, a cultural barrier might also be at play. More specifically, Ko and Koh [20] explained how not seeking help among Korean nurses and older people was influenced by cultural norms. The study also revealed that most of the elderly might be ashamed of reporting and seeking help thinking that doing so would let them to teach discipline to their children. Additionally, nurses thought that it would not be appropriate to interfere in a private matter.

As suggested by previous studies, there is a positive relationship between training and education for nurses in elder abuse, and reporting and reduction in cases [3,6,20,21]. On this note, there is evidence to suggest that participants who were victimized would prefer a specialized nurse in detecting and managing their case [22]. Additionally, Teresi et al. [21] suggested that providing training and education to nurses would help achieve the quality of life for the elderly. Moreover, education would increase the assessing or identification skills of nurses in elder abuse cases. Since many studies have highlighted the importance of education and training for nurses in identifying and managing elder abuse, this study aims to analyse the trainings tailored for nurses that provided education in elder abuse. The aim of this literature review is to explore what is the effectiveness of a nurse training program in elder abuse management and which training methods are more efficient.

## 2. Methodology

This systematic review discusses articles that assess the effectiveness of the educational/training programs in elderly abuse and thus it can be classified as an effectiveness review according to [23]. In order to ensure the reliability and validity of this study, we followed Joanna Briggs Institute’s (JBI) guidelines for reviewing the quality of articles included. We formulated specific research questions in order to focus the review’s search strategy and sampling.

### 2.1. Research Questions

The research questions of this review were as follows:

- What is the effectiveness of a nurse training program in elder abuse management?
- What is the efficiency of different training methods about elder abuse?

### 2.2. Search Strategy

A comprehensive and systematic review was conducted using the guidelines set forth in the Preferred Reporting Items for Systematic Reviews and Meta-Analyses (PRISMA) statement [24]. The electronic databases used were Medline, CINAHL, and Health Source from period 2010–2022. The following keywords were used alone or in combination: “Education’’, “Training’’, “Nurses’’, “Elder Abuse’’, “Mistreatment’’, “Effectiveness”. Table 1 shows the inclusion and exclusion criteria used for selecting the relevant articles for this review.

### 2.3. Critical Appraisal

All studies that met the inclusion criteria were evaluated by two independent reviewers on their quality using Joanna Briggs Institute Qualitative Assessment and Review Instrument Critical Appraisal Checklist for Studies Reporting Prevalence Data—Results, for Randomized Controlled Trials-Results, for Quasi-Experimental Studies and for Analytical Cross-Sectional Studies. As per Table 2, these are checklists commonly used to assess the methodological quality of a study and to determine the extent to which a study has addressed the possibility of bias in its design, conduct and analysis [25].

### 2.4. Data Extraction

The extracted data included authors, title, year of publication, methodology aims/purposes, sample, instruments, and findings. The data extraction was implemented by two researchers and was checked for accuracy by a third researcher.

### 2.5. Data Synthesis

Content analysis was used to synthesize the data in order to analyze the text of stories for their implicit meanings. According to Mikkonen and Kääriäinen [29] content analysis can be beneficial to summarising the key elements in the large amount of data identified during the review process. For the current review, we followed the process proposed by Zhang and Wildemuth [30] to categorize in themes the effectiveness of nurse training programs in elder abuse.

## 3. Results

**Figure 1:**
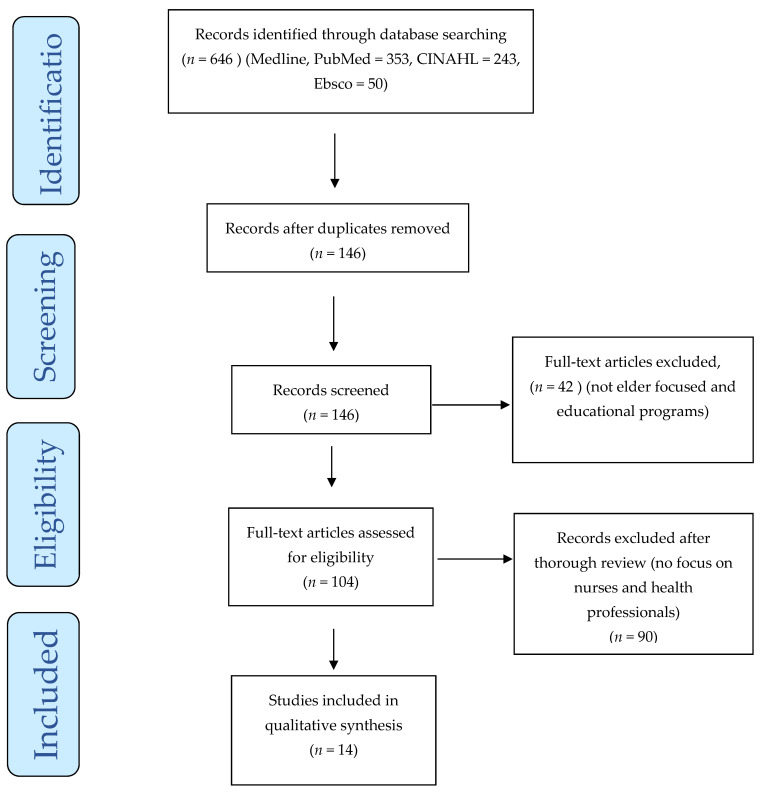
PRISMA flowchart with the search strategy of the systematic review.

The search procedure initially generated 646 articles that related to elder abuse. As per the PRISMA flowchart in Figure 1 below, all the articles, title, methods, intervention, and participants were double-checked, and all the duplicate and irrelevant articles were removed from the study. As a result, 146 articles were included for further screening. Forty-two (42) articles were not relevant to nurses’ trainings and were removed. The remaining 104 articles were reviewed thoroughly by two researchers independently. This thorough review process resulted in excluding another 90 articles which did not meet the inclusion criteria. A total of fourteen (14) articles that met the inclusion criteria were included in this literature review and the results are analysed further below.

## 4. PRISMA Flow Diagram

This research reviewed 14 articles, studied in different geographical areas, which evaluated the impact of education about elder abuse on nurses and healthcare professionals. Some of the studies have used training modules and educational training strategies developed before and some have developed a new teaching module and evaluated its effectiveness. As per the critical appraisal in Section 2.3, there were five randomized control trial studies [3,8,21,26,27], six quasi-experimental study, [1,4,6,16,17,28] and three Cross-Sectional studies [5,9,20]. All of them are listed in more detail in Table 3 below.

Reviewing the articles qualitatively, five themes derived, namely knowledge enhancement; increased identification of elder abuse; reporting of elder abuse; improved competency in dealing with elder abuse; and training methods. Each theme is analysed below.

### 4.1. Knowledge Enhancement

Several studies have explored the importance of training and education for nurses in dealing with cases of elder abuse [4,5,8,21]. Research evidence has shown that educational training has enhanced the level of knowledge about elder abuse among nurses and other healthcare professionals [3,4,8,27]. More specifically, Ko and Koh [20] explained that on average nurses’ knowledge of elder abuse was only 3.74 out of 10 and that 18.6% of the nurses were not willing to report the suspected case. Interestingly, the study showed a relationship between willingness to report and knowledge of elder abuse, which explained that nurses could not report due to the lack of knowledge in assessing elder abuse. As noted in the findings, nurses were not aware that elder abuse was taking place, and this was due to a lack of knowledge [20]. This shows the importance of having the knowledge to deal with an elder abuse case.

Teresi et al. [21] showed that following a post-educational training participants’ knowledge of elder abuse increased. Another study by Ross et al. [4] noted that after providing education through a lecture, simulation with standardized patients, and debriefing the score on knowledge among the participants increased by 28%. Furthermore, in a six-month prospective study to evaluate the impact of intensive training on the identification and management of elder abuse, the knowledge among the participants in the intervention group increased by 40% after the training [3]. Along similar lines, Ejaz et al. [28] found that online training in elder abuse had a significant positive impact on developing participants’ knowledge. Additionally, the results showed that the training was beneficial in improving the care provided to older adults [28]. Furthermore, Mont et al. [16] revealed that participants’ overall self-reported knowledge in elder abuse increased significantly following a training session. Like in other studies, the training increased participants’ competence level in dealing with the elder abuse situation [16]. Ross et al.’s [4] study discussed similar results by describing how participants’ knowledge related to elder abuse increased after receiving educational training through lectures, simulation, and debriefing, since their knowledge score increased from 4.71 to 6.02 [4]. The participants in this study stated that after receiving the training their knowledge, skills, and self-efficacy in identifying the elder abuse case improved [4]. Ejaz et al. [28] measured the impact of an online training on the background of elder abuse, screening of elder abuse, and reporting protocol for cases of elder abuse, and found that participants’ knowledge and competence improved. Interestingly, Estebsari et al. [26] showed that the frequency of acquired knowledge of elder abuse, self-efficacy, health-promoting behaviour, and risk of elder abuse was statistically similar in both intervention and control groups [26]. However, after the training the intervention group’s knowledge was slightly higher than the control group’s [26].

The importance of knowledge in dealing with cases of elder abuse was highly emphasized in other studies [1,5]. In general, research has shown that nurses with knowledge were able to recognize the elder abuse case more frequently than the ones who no or poor knowledge [1,3,21].

### 4.2. Increased Identification of Elder Abuse

As a result of improved knowledge, research studies have shown that educational training has increased nurses’ ability to identify and recognize the occurrence of elder abuse. As noted already, Ko and Koh [20] discussed that nurses’ knowledge of elder abuse was 3.74 out of 10 and that 18.6% of the nurses were not willing to report the suspected case. Furthermore, the study showed a relationship between willingness to report and knowledge of elder abuse, which explained that nurses could not report due to a lack of knowledge in assessing elder abuse [20]. Teresi et al. [21] revealed that after receiving training in elder abuse, the recognition ability among nurses improved significantly. Teresi et al.’s [21] study showed a direct connection between educating staff and increasing their ability to identify elder maltreatment as the identification rate in the experimental group was six times higher than in the controlled group. Likewise, another study done among Italian nurses and nursing students noted that nurses with working experience and students who were trained in dealing with elder abuse were able to correctly identify most of the abusive cases [9]. However, neglect was only identified by 25% of nurses and 20% of students, which indicated the need for deeper education training in such topics [9]. Harries et al. [8] measured the effectiveness of training given to novice and expert healthcare workers in improving financial abuse detection and yielded similar findings as previous studies. In this study participants who received the online training, their mean score on the certainty of risk of abuse in the case scenario increased post-training and were more confident in identifying the risk of elder abuse. Likewise, the identification score among the novice who was in the intervention group was higher compared to the controlled group [8]. The authors noted that before gaining any kind of training or information the novice were not confident or not even aware that abuse was occurring. After the training, the ability of novices to detect abuse increased. However, the novices were not able to match the level of detection of the experts, and this is because the novice were not as experienced as the experts were. Similarly, another educational intervention given to the nurses was able to decrease the prevalence of elder abuse [1]. Along similar lines, Ross et al. [4] found that participants were able to identify and assess elder abuse cases confidently after they received training. In the open comment box, students mentioned that they felt confident in identifying signs of abuse post-test. Furthermore, the findings of Ghaffari et al., [27] supported previous studies by indicating that the participants were able to recognize the elder abuse case 36% more after receiving educational training. However, one study noted that participants were not able to identify all types of abuse even after receiving training [1]. This study is similar to research conducted by Ejaz et al. [28], where participants did not show higher improvement in the screening of abuse even after the training was provided. These two articles noted that even after receiving training participants’ scores in the identification of elder abuse did not increase, which suggested the need for thorough evaluation of the training module [1,28].

### 4.3. Reporting of Elder Abuse

Reporting the occurrence of elder abuse cases is crucial for developing prevention strategies, proper management, and educational trainings. A study by Harries et al. [8] showed that only 14% of financial abuse on the elderly was reported; however, the actual number of cases was higher. There was a study done on Korean nurses’ willingness to report elder abuse cases, in which many nurses were not willing to report cases [20]. The findings revealed that participants were not aware of the reporting process which showed that the institution was not able to provide information and training in elder abuse [20]. Teresi et al. [21] showed that staff-reported Resident-Resident Elder Mistreatment (R-REM) cases were equal in both control and intervention before receiving education about elder abuse. After receiving education, the nurses were able to report incidents seven more higher than that of the control group. This explains the significant role that training and education play in increasing reporting which later helps in implementing and developing strategies to improve dealing with elder abuse. The authors clarified that education might give an incentive to document the incidence [5,21]. Along similar lines, other studies revealed that reporting on elder abuse increased after attending educational training that consisted of lectures, simulation lab, and debriefings [4,6,17,26,28]. Additionally, participants stated that after receiving the education they felt confident in reporting the cases of elder abuse [4]. After attending the training on elder abuse education, participants were able to assess, identify and report the elder abuse incidents confidently [16].

It is pivotal to know about elder abuse to be able to document and report the case. If a nurse or healthcare professional is aware of what causes elder abuse, how to identify it, and what to do once they identify it, then they are willing to report more frequently. As shown in many studies, only after gaining knowledge about elder abuse, its characteristics, and how to report and manage it, participants increased reporting significantly [4,8,16,21,27]. Interestingly, Ko and Koh. [20] showed that nurses were not willing to report elder abuse incidents they noticed because they were not aware of the process after reporting, and the mandatory law to report the incidents. This clearly explains that when someone is not well informed of the process of reporting or does not have knowledge of elder abuse cases tend to go unreported, causing more harm to the elderly.

### 4.4. Improved Competency in Dealing with Elder Abuse

Competency in dealing with elder abuse is associated with relevant training. That is, Harries et al. [8] showed that participants who were experienced in detecting financial abuse were more confident in dealing with and identifying cases of elder abuse. Additionally, the authors indicated that the novice participants, after the training, were more competent in recognizing the incidents since their identification rate increased significantly [8]. This result is similar to a study by Ross et al. [4], which revealed that participants from the intervention group identified a case of elder abuse more competently than from the control group. Additionally, in this study, nursing students who were given a lecture and case scenarios to identify incidents of abuse and abusive strategies, were more likely to deal with cases more accurately. Additionally, Ko and Koh [20] showed that nurses had significant improvement in their perception of caring for abused older adults as the mean score increased from 2.9 to 3.06 post-training. Furthermore, nurses’ perceived knowledge and competence about the content of elder abuse increased in all core domains. More specifically, identifying abuse (pre-training Mean 3.9 vs. post-Mean 4.4), documentation, legal, and legislative issues (pre-training M: 3.2 vs. post-M: 4.1), interviews with an older adult, caregiver, and other relevant courses (pre-training Mean: 3.7 vs. post. M: 4.2), initial assessment (M: 3.6 vs. M: 4.3), medical and forensic examination (M: 3.9 vs. M: 4.3), case summary, discharge plan, follow-up care (M: 3.6 vs. M: 4.2). Likewise, participants who had debriefing and case studies during their intervention, reported that they were more confident in applying their knowledge about elder abuse [4]. Moreover, Pickering et al. [17] discussed that participants were able to confidently identify the incidence of elder abuse that needed to be reported and they were able to correctly answer the questions, which showed their competency in dealing with elder abuse effectively. Finally, Kosa et al. [6] noted that nurse’s level of competency increased significantly from Mean 1.1 to Mean 3.8 post-training.

### 4.5. Training Methods

The review of selected articles showed that different teaching methods yielded different results. For example, Mydin et al. [3] suggested that the use of mixed educational trainings for nurses were more effective than only a face-to-face training alone. In Teresi et al.’s [21] study, participants were provided training through a power-point presentation and discussion, which had a positive impact the participants’ learning ability. This study also included a film about elder mistreatment. Interestingly, due to the inclusion of a case scenario in the film participants were able to better understand the theoretical knowledge gained during lectures and then visualized it through the film, which increased their identification skills more than in the controlled group [21]. Similarly, Mydin et al. [3] reported that the participants who attended intensive training programs along with educational videos increased their recognition ability more than the participants who only attended the intensive training program.

Additionally, other studies included lectures, PowerPoint presentations, and booklets [17,26,27]. For example, in Ghaffari et al.’s [27] training involved face-to-face in a classroom, which included visual presentation through the PowerPoint, plus they gave booklets that participants would be able to look at their own pace. The finding of this study suggested that participants who were in the intervention group had increased recognition skills by 35.88 scores higher than the control group. These results explain that the use of combined educational training is beneficial for better understanding and remembering course material.

Pelotti et al.’s [9] studied a programme which used only face-to-face lecture to train participants in elder abuse. Participants were not able to identify all the abusive strategies toward the elderly, especially neglect [9]. However, the participants who had previous experience in taking care of the elderly were able to identify more abusive strategies.

Interestingly, the inclusion of e-learning assisted participants in revising their study materials, resulting in improving their knowledge and identification skills. One of the articles utilized an online learning module, in which the majority of the participants reported that they were satisfied with their learning [17]. Online and mobile-based teaching methods, rather than long lectures, were easy to use, and participants would be able to complete their tasks at their own pace which helped enhance learning [6]. Therefore, a few studies used lectures along with providing booklets, and educational videos for revision. Harries et al. [8] provided written materials, graphical descriptions, case scenarios on how to identify elder abuse, and what cues to look at assessing for elder abuse. This combination of methods resulted in a positive impact on the frequency of identifying cases of elder abuse. Similarly, Ross et al. [4] explained that participants were able to gain knowledge and recognize more incidents of elder abuse when attended lectures and were involved in standardized patient simulation. The participants in this study explained that the use of standardized patient simulation helped them to work with realistic scenarios, which increased their identification skills [4]. In support, Mont et al. [16] discussed that inclusion of discussion strategies post educational face-to-face lectures has shown a positive impact on nurses’ identification skills.

The use of debriefing and discussion was also highly appreciated in many studies [3,4,16]. More specifically, Pickering et al. [17] emphasized that debriefing and focused feedback during the intervention were positive points in increasing participants’ recognition skills and knowledge of elder abuse. It seems that debriefing helps participants to reflect on actions taken, which can further enhance the knowledge and understanding of the best action to be taken. Additionally, having a discussion that supports participants to look and understand through another person’s point of view help them to understand other options that can be used to deal with elder abuse. Therefore, the most useful teaching methods in the articles reviewed were lecture, power-point presentation, case study, simulation, debriefing, discussion, e-learning, and educational videos. Studies showed that combinations of these methods have increased the nurse’s ability to identify elder abuse more frequently after receiving the training. In conclusion, carefully designed and delivery of training delivery is very important for enhancing nurses’ knowledge in elder abuse and increasing identification and reporting incidents of elderly’s mistreatment.

## 5. Discussion

This systematic review has collectively reviewed the literature that has studied the impact of education among nurses on identifying and dealing with elder abuse. The findings of this review revealed only 14 trainings in elder abuse tailored for nurses in the last 10 years. Out of the 14 articles reviewed 13 articles evaluated the impact of educational intervention directly among nurses in increasing their ability in identifying elder abuse. The finding of the remaining article identified a link between the nurses’ knowledge and their willingness to report an incident [20]. The reviewed articles indicated that after the educational intervention nurses’ knowledge of elder abuse increased and the frequency of incident identification and reporting improved significantly [3,4,8,9,21]. 

Studies have noted that the identification rate decreased in three- and six-month period after the training in two different studies [3,27]. This shows the importance of long-term and continuing educational training for nurses. Providing long-term and continuing education could help nurses to better familiarize with the issue of elder abuse. Moreover, the use of real-time cases that have occurred at the workplace could help in enlightening nurses’ knowledge about elder abuse. Additionally, when nurses are been given education more often and continuously, they are more likely to catch up missed information from previous training. Every new training could reinforce increasing nurses’ knowledge about elder abuse which is beneficial in dealing with elder abuse cases. Thus, health care institutions need to evaluate their staff’s educational status and provide training based on their needs, with a long-term continuing intervention. Providing timely educational training with interval of 3 months to 6 months was found to be more effective. Therefore, health care institutions should provide educational training every three to six months depending on staff’s knowledge.

Different studies used combined teaching delivery methods such as lecture, simulation, debriefing, discussion, PowerPoint presentation, and educational video [3,4,6,21]. The result of these studies showed a greater positive impact on nurses’ knowledge, increased identification, and increased reporting. The use of face-to-face lectures and booklets or educational videos for revision enhances nurses’ understanding of elder abuse. Additionally, the use of debriefing and discussion assists nurses in reflecting on the cases and helps to see through another angle which can positively affect how they might deal with elder abuse situations another time. Furthermore, the use of simulation with a standardized patient is very important for having a realistic view of the case scenario which assists in better learning how to identify elder abuse. Therefore, while providing educational training to nurses, the use of a combined education delivery methods is important.

In spite of the findings showing the effectiveness of trainings for nurses, the reviewed studies have revealed some importance limitations in terms of the long-term positive outcomes regarding prevention of elder abuse. More specifically, although trainings have improved skills in identifying and reporting incidents of elder abuse, rates of abuse remain high. This shows that trainings can enhance awareness, confidence and general competence but they do not solve or ameliorate the problem. It seems that any trainings should be part of a wider policy plan, which should reach out to encompass other structures of influence, such as creating a culture of acceptance, value and respect of the elderly in society, and the provision of high-quality care that would be under constant review and evaluation. High-quality care for the elderly should be accompanied by structural procedures within healthcare settings. For instance, management teams of healthcare settings should ensure that there are procedures in place regarding training, reporting, and caring of cases of elder abuse, with clear pathways of investigating and decision making on reported complaints. Moreover, healthcare institutions can increase the use of available networking and technological services to make it more approachable for nurses to study or get training, such as the use of mobile-based training, and telegram applications that facilitate effective interactions [1]. For nurses who cannot attend any training due to personal reasons, this type of training service would be more beneficial. The use of mobile-based training would encourage nurses to gain knowledge at their own pace as well. Finally, management teams need to be more vigilant about the condition of elder abuse in their work environment. They can run weekly check-ups of the reported incidents, doing a follow-up on cases, and also having debriefings of the case with the nurses. This could help in finding other solutions to preventing elder abuse and gaining knowledge of the situation.

**Figure 2:**
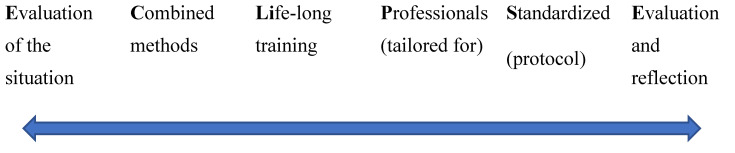
The ECLiPSE pathway for effective training of nurses in handling elder abuse cases.

Based on the results of this systematic review and the limitations of the studies reviews as presented above we propose the ECLiPSE pathway for the effective training of nurses to handle elder abuse cases, eventually contributing to decreasing the incidents. As per Figure 2 below, ECLiPSE stands for **E**valuation of the situation which means that nurses’ knowledge, skills and experiences as well as the prevalence of elder abuse should be evaluated first before any trainings are scheduled. This is important to do in order know in advance where the gaps are and how the trainings should be designed. The trainings should not rely on one method but on a **C**ombination of methods in order to achieve the best results. Such combination would help with enhancing knowledge and skills by reflecting on the learning styles of the trainings. Any trainings should be **L**ife-long and not ad hoc or one-off sessions. It has been well documented in education that continuous education in a spiral fashion (revisiting material but in different contexts) improves learning. Training should be tailored for Professionals and be Standardized, with a training protocol, so that all nurses acquire similar knowledge and skills, and follow the same procedures. This will contribute to creating a culture of reporting and handling cases of elder abuse. Such standardization would also help communication among the nurses as well as between the older patients and nurses regarding issues of abuse and mechanisms of reporting and handling. Finally, all trainings and outcomes should be **E**valuated and reflected upon in order to assess their impact and improve them. Although, this is the final step, it is also the beginning as the arrow shows in Figure 2 below. That is, through evaluation and reflection all steps should be revisited and revised in order to ensure the best possible outcome. Apart from a pathway of action and implementation plan, ECLiPSE phonetically resembles the word “eclipse” to symbolize a way to reduce or help the elder abuse cases to extinct or decrease the number of untreated and mistreated cases of elder abuse.

In addition to the ECLiPSE pathway for training purposes, this systematic review is an important contribution to scholarship as it has shown that the positive impact of education among nurses in increasing their knowledge, identification skills, and reporting of cases. However, there is a need for more research studies to be conducted in different geographical areas, evaluating the effectiveness of developed educational training curricula and programs. The following research questions or areas of research have derived from this systematic review:

- Which trainings are more effective and why?
- What types of elder abuse require more focused training?
- How should the topic of elder abuse and mistreatment be integrated in nursing curricula? What is the effectiveness of such integration?
- What is the impact of nurses’ training on long-term reduction in the prevalence of elder abuse?
- What other changes need to be made structurally, politically, and culturally in order to significantly decrease the rates of elder abuse?
- Which other groups, apart from nurses, need to be trained in order to decrease prevalence of elder abuse?

## 6. Conclusions

Based on a systematic review of 14 articles, this study has shown that the available training of nurses in identifying, reporting and handling cases of elder abuse improve knowledge, skills, competence, and confidence, with a potential positive impact on long-term health and wellbeing of older adults. The study revealed that training per se is not enough and that systematic, continues and life-long training is much more effective than one-off or ad hoc interventional programmes. Finally, the type of training matters as a combination of methods (e.g., lectures, scenarios, e-learning etc) yielded better results and helped trainees to retain acquired knowledge and skills over longer period of time. In order to provide a clear pathway of effective training of nurses we propose ECLiPSE which stands for Evaluation of situation, Combined methods, Life-long training, Standardized training and Evaluation and reflection. This study has also opened new directions in research by placing emphasis on the importance of life-long and standardized training as well as of the impact of training on the reduction in incidents. It seems that there is a need for the development of a training protocol which can be used across universities, health institutions, healthcare systems and countries.

## Figures and Tables

**Table 1:** Inclusion and exclusion criteria.

**Inclusion**	Primary research articles or chapters
	Theses and dissertations
	Literature reviews and meta-analyses
	Conference research papers
	Studies of nurses’ trainings and elderly abuse Published in English Period of publication 2010–2022
**Exclusion**	Research articles, chapters, reviews, theses or dissertations, conference research papers which do not relate to nurses’ trainings and elderly abuse
	Any of the above publications published before 2010
	Published in languages other than English

**Table 2:** Critical Appraisal Checklist.

**JBI Critical Appraisal Checklist for Randomized Control Trials.**													
**Reference**	**Q1**	**Q2**	**Q3**	**Q4**	**Q5**	**Q6**	**Q7**	**Q8**	**Q9**	**Q10**	**Q11**	**Q12**	**Q13**
[21]	√	√	√	No	√	√	√	√	√	√	√	√	√
[8]	√	√	√	No	√	√	√	√	√	√	√	√	√
[26]	√	√	√	No	√	√	√	√	√	√	√	√	√
[27]	√	√	√	No	√	√	√	√	√	√	√	√	√
[3]	√	√	√	√	√	√	√	√	√	√	√	√	√
**JBI Critical Appraisal Checklist for Quasi-Experimental Studies.**													
**Reference**	**Q1**	**Q2**	**Q3**	**Q4**	**Q5**	**Q6**	**Q7**	**Q8**	**Q9**				
[6]	√	√	√	No	√	√	√	√	√				
[1]	√	√	√	√	√	√	√	√	√				
[4]	√	√	√	No	√	√	√	√	√				
[16]	√	√	√	√	√	√	√	√	√				
[17]	√	√	√	No	√	√	√	√	√				
[28]	√	√	√	No	√	√	√	√	√				
**JBI Critical Appraisal Checklist for Analytical Cross-Sectional Studies.**													
**Reference**	**Q1**	**Q2**	**Q3**	**Q4**	**Q5**	**Q6**	**Q7**	**Q8**					
[9]	√	√	√	√	√	√	√	√					
[5]	√	√	√	√	√	√	√	√					
[20]	√	√	√	√	√	√	√	√					

**Table 3:** Research which evaluated the impact of education about elder abuse on nurses and healthcare professionals.

Reference	Methodology	Sample	Results
[20]	Cross-Sectional studies	365 nurses	The sample consisted of 359 female and 6 male nurses. 18.6% of nurses were not willing to report suspected elder abuse. Reason: 50.0% Considering it family matter due to Korean tradition influence not intervening in family discussion: 16.2% were not certain of the reporter’s identity protection; 11.8% thought it was not issue could be solved by legal process. The mean score of knowledge of elder abuse law was 3.74 (SD ¼ 1.83) on a scale of 0 to 7. Nurses willing to report were trained and had knowledge on elder abuse law. The years of experience, knowledge on elder abuse, and perceived severity were significant predictors of willingness to report the case. The nurses with knowledge of elder abuse had 1.27 times higher the willingness to report the case.
[21]	Randomized control trial	1405 residents 325 nurses	Staff on the experimental units received training and implementation protocols and Staff on the comparison units did not Experimental Group Knowledge: nursing staff’s gain in knowledge was evidenced for both Module 1 (*p* < 0.001) and Module 2 (*p* < 0.001) in that the total post-test scores for each module were significantly different (indicative of higher levels of knowledge) from the total pre-test scores. After training experimental group recognized and reported twice as many incidents as the control group. (6 times higher than the controlled group) Higher levels of recognition and documentation of resident-to-resident mistreatment (R-REM) were observed in the experimental group. At six months the recognition and reporting in the experimental group increased seven times higher. Additionally, 10 times higher in 12 months. The average reported events per resident per year was 0.35 in control group and 2.06 were reported from intervention group.
[9]	Cross-Sectional studies	193 nursing students 76 nurses	Neglect was identified by 25% nurses and 20% of students. Locking someone at home was identified half of them (18 nurses, 39 nurses), majority labelled it as bad idea but did not recognise as abusive. Most of the nurses and student identified non-abusive strategies. Corelation between education and work experience was found. The nurses were able to recognise abusive strategies more accurately. This was seen in relation to their work experience and the education received. 74 nurses and 184 student received education on elder abuse, 88 nurses worked for elderly, 53 taught what to do when elder abuse was noticed, who identified abusive strategy of putting table on lap to limit the mobility of elderly.
[8]	Randomized control trial	Novice health and social care professionals Intervention—78 (Online web-based decision training Control = 76 (no training)	154 novices made judgements of risk of abuse (“certainty of abuse”) on the same set of 43 scenarios that the experienced clinicians had judged. The experts mean’ certainty of abuse was higher than of novice pre-intervention in 28 scenarios. The experts have consistency in their detection. Detection of financial abuse the scores of the control and intervention Control Group: Pre-test—0.45 and post-test—0.55 Intervention Group: Pre-test—0.45 and post-test—0.71 Expert were more certain than the novice in detection of financial abuse (Mean 70.61 vs. 58.04). The intervention group mean score (64.84) than control group (61.41) post-test in certainty of identification. The intervention group were sensitivity in saying at risk for incident was not more than control group pretraining. Post-training, the intervention group were more sensitive in stating risk of abuse.
[28]	Quasi-experimental study	Nurse, social worker, counsellors, nurse practitioners. (453)	It was composed of three modules covering background, screening, and reporting abuse. Of 453 enrolees, 273 completed at least one module and 212 completed all three. Pre- and post-training surveys for each module were used to examine changes in the proportion of correct answers for each question, using the related-samples Cochran’s Q statistic. Module 1: Knowledge on background on abuse significantly increased. (4 question: correct pretraining vs. post-training 29% vs. 78% 48% vs. 67% 54% vs. 73% 83% vs. 90% Module 2: Knowledge on screening of abuse Correct pretraining vs. post-training 78% vs. 83% 38% vs. 43% 85% vs. 84% Module 3: Reporting protocol for cases of abuse 14% vs. 63% 41% vs. 88% 74% vs. 69% 74% vs. 90%
[16]	Quasi-experimental study	from 18 SANEs. Sexual Assault nurse examiner	A 52-item pre- and post-training questionnaire was administered that assessed participants’ self-reported knowledge and perceived skills-based competence related to elder abuse care. A curriculum training evaluation survey was also delivered following the training. Overall, Knowledge: Self-reported knowledge/expertise on elder abuse increased (2.36 pre vs. 3.45 post). Knowledge and Skill-based Competence: Increase in all six domain- Older adults and abuse (3.53 vs. 4.61), Documentation, legislation, legal issues (2.70 vs. 4.17), Interview with older adults, caregiver, and other relevant contacts (3.40 vs. 4.24), Assessment (3.28 vs. 4.17), Medical and Forensic Examination (3.83 vs. 4.41), Case summary discharge plan, follow-up care (3.37 vs. 4.04). There was increase in knowledge on 49 items out of 52 post-training.
[17]	Quasi-experimental study	39 nurses from the introductory 101 course a 36 staff participated in virtual-reality-based advanced training	After virtual-reality-based advanced training: Participants appreciated the interactive nature of the training, receiving feedback in real-time, debriefings focused on clinical reasoning and the QualCare Scale. Participants were usually able to obtain the required information. The decision level regarding whether to report potential Elder Abuse and Neglect (EA/N), decisions had 99% accuracy. About specific changes implemented in practice revealed participants implemented a variety of changes including being more thoughtful about: the implications of the living environment, probing more about resource distribution, more detailed assessment of nutrition and food access, and utilizing questions from the human rights assessment subscale to better understand the relationship dynamics
[26]	Randomized control trialG	464 participants	The demographic characteristics between intervention and control group were similar. The frequency of knowledge of elder abuse was similar pre training in both control and intervention (113 vs. 177). The knowledge, self-efficacy, health promoting behaviours increased post-training in intervention group compared to control (220 vs. 108) In logistic model, post- training social support showed connection with intervention, health status. The intervention did not directly help to prevent the risk of elder abuse but helped with other variables to prevent it.
[3]	Randomized control trial	203 primary-care nurses	Baseline knowledge on Elder Abuse and Neglect (EAN), Controlled—4.49 Intervention—4.66(lecture) 4.51(video) There was increase in Knowledge after intervention: Control—4.96 Intensive Training Program (ITP)—6.50 ITP+ - 6.44 Attitude: Control: 20.91 ITP: 22.61 ITP+: 23.00 Confidence Control: 19.10 ITP: 21.72 ITP+: 22.75 Post-intervention There was increase in the knowledge, attitude and confidence to intervene in EAN among participant of ITP and ITP+ group compared to controlled group
[4]	Quasi-experimental study	119 students enrolled in the gerontology course were the participants	There was a significant difference in pre-test and post-test knowledge (4.71 vs. 6.02) and skills (3.87 vs. 4.52). Attitude even pre-test was 4.9 among students (max 5), so there was no space left for improvement. Training on how to identify, assess, and report elder abuse enhanced students learning. Lecture with M = 4.46, Simulation (M = 4.68), Debriefing (M = 4.68). There was comment space for students post-training, they stated that lecture, simulation, and debriefing increased their knowledge, skills, and self-efficacy in preventing, identifying, and advocating for victims of elder abuse.
[1]	Quasi-experimental study	88 nurses were given training 431 elderly people were assessed with questionnaire	Characteristics: Mean age 71.68 years before and 73.33 years after interventions. The mean score on elder abuse was 29.16 before and 32.62 after which means that elder abuse occurrence was reduced. The highest subscale was scored by psychological abuse, which mean it was the lowest level of abuse among other type of abuse. The score of psychological abuse before intervention was 4.34 and 4.56 after intervention. The physical abuse before intervention 3.84 and 4.67 after intervention. (Identified more as it is more objective). Neglect was identified as least prevailed among others. Education reduced perceived abuse among hospitalised older adults
[27]	Randomized control trial	120 nurses Intervention group—60 Controlled—60. Intervention—Received education via power point lectures, education booklets.	21.7% participant experience at least one case of elder abuse (EA). 55.8% believed the EA reporting is not their responsibility. No significant difference in general characteristics. Baseline recognition ability score: controlled—224.9, intervention: 220.4. After intervention: Controlled 224.85, intervention255.96. After intervention, staff recognition skills increased. The highest score post intervention was related to physical component. Participants noted that elderly’s dependency on caregiver had high risk in occurrence of Elderly Abuse (EA) especially in case alcohol and drug dependency. There was slight decrease in score from 1 month to 3 month which suggest that continuing education is required.
[5]	Cross-Sectional studies	324 nursing students	Characteristics, 90.3% female and rest male. 13.8% only received elder abuse education. 3.1% witnessed Elderly Abuse, 1.5 % psychological, 2.8% neglect, 2.8% reported. Level of importance on education was higher than of performance in elder abuse identification (4.29 vs. 3.08). Importance and performance-based score on educational topic: physical 4.49, psychological 4.47, understanding of physical an emotional change 4.1, human rights 4.40, neglect 4.43. Certain items have highest value in performance than in importance.
[6]	Quasi-experimental study	54 nurses	Significant increase in expertise in caring for abused elderly after intervention Knowledge and competence score increase from Pre-M 3 to post Mean 4.4 on elder abuse care. Documentation, legal and legal issuers knowledge raised from 3.2 to 4.1 Initial assessment 3.6 vs. 4.6 Participant were satisfied with the curriculum: Clarity (90.6%), ability of material to engage and keep attention by 76.5%, Knowledge and management by 90.4%.
